# The relation between finger gnosis and mathematical ability: why redeployment of neural circuits best explains the finding

**DOI:** 10.3389/fpsyg.2013.00877

**Published:** 2013-12-05

**Authors:** Marcie Penner-Wilger, Michael L. Anderson

**Affiliations:** ^1^Department of Psychology, King’s University College at Western UniversityLondon, ON, Canada; ^2^Department of Psychology, Franklin & Marshall CollegeLancaster, PA, USA; ^3^Institute for Advanced Computer Studies, University of Maryland, College ParkMD, USA

**Keywords:** number representation, finger representation, neural substrate, exaptation, function–structure mapping, cross-domain modeling

## Abstract

This paper elaborates a novel hypothesis regarding the observed predictive relation between finger gnosis and mathematical ability. In brief, we suggest that these two cognitive phenomena have overlapping neural substrates, as the result of the re-use (“redeployment”) of part of the finger gnosis circuit for the purpose of representing numbers. We offer some background on the relation and current explanations for it; an outline of our alternate hypothesis; some evidence supporting redeployment over current views; and a plan for further research.

## INTRODUCTION

Finger gnosis, alternatively called finger recognition or finger localization, is the presence of an intact finger schema or “finger sense” (Gerstmann, 1940). A variety of neuropsychological tests have been designed to assess the presence of finger gnosis, or its absence – finger agnosia – in neuropsychological populations. In one common test (Baron, 2004), the examiner shields the participant’s hand from view and lightly touches one or more fingers. The participant is asked to identify which fingers were touched. Finger gnosis tests have been used by neuropsychologists to provide an indication of parietal lobe damage (Gilandas et al., 1984). Finger agnosia is one of a constellation of symptoms in Gerstmann’s syndrome, along with acalculia, agraphia, and left–right disorientation. Gerstmann (1940) identified finger agnosia or the loss of “finger sense” as the core deficit of the syndrome.

Perhaps surprisingly, recent research is demonstrating links between finger gnosis and mathematics ability in neuropsychologically normal children. For instance, Fayol et al. (1998) discovered that a set of neuropsychological tests, including tests of finger gnosis, was the best longitudinal predictor of Grade 1 children’s math scores. This finding was confirmed by Noël (2005), who demonstrated that children’s finger gnosis scores predicted accuracy and fluency on a variety of mathematical tests, both concurrently in Grade 1 and longitudinally one year later. University students’ finger gnosis scores also predict calculation skill, concurrently (Penner-Wilger, 2013; Penner-Wilger et al., in preparation).

Penner-Wilger et al. (2007) and Penner-Wilger et al. (submitted) found that finger gnosis was related to children’s number system knowledge and calculation skill concurrently in Grade 1. Moreover, Penner-Wilger et al. (2009) found that finger gnosis in Grade 1 predicted performance on tasks designed to assess number representations – number comparison and estimation – in Grade 2. Thus, there is converging evidence for a relation between finger gnosis and math ability in both selected and typically developing populations and evidence to suggest that this relation is mediated by number representations.

Though a clear relation has been demonstrated between finger gnosis and math, what remains unclear is what the true nature of the relation is. It is possible that the true underlying relation is correlational, as proposed by Dehaene et al. (2003). Alternatively, as proposed by Butterworth (1999), the relation may be directly causal. As a result of our cross-domain modeling approach, we propose a third option, that the underlying relation between finger gnosis and math ability is indirectly causal, the result of a neural resource shared by finger representation and number representation, among other tasks.

In the following sub-sections we first outline the two prevailing views, termed localizationist and functionalist (Noël, 2005), and briefly review some of the evidence for and against these views. This is followed by the introduction of a third view – the redeployment view – that explains the relation in terms of a shared brain region that contributes to both relata. Because the redeployment view will be less familiar to readers, we provide a much more detailed account of it than we do of the other views. Section 2 is devoted to a discussion of the available evidence, which appears on balance to favor the indirectly causal redeployment view.

## THREE VIEWS OF THE RELATION BETWEEN FINGER GNOSIS AND MATH ABILITY

### LOCALIZATIONIST VIEW

On the localizationist view, finger gnosis is related to math ability because the two abilities are supported by neighboring brain regions in the parietal lobe (Dehaene et al., 2003). The comorbidity of finger agnosia and acalculia, as seen in Gerstmann’s syndrome, is explained as arising from common vascularization to the associated parietal areas, with damage typically affecting both areas. The relation between finger gnosis and math in typically developing children is a reflection of the correlated developmental trajectories of neighboring brain regions. Consistent with the localizationist view, Simon et al. (2002) found regions in the intraparietal sulcus activated for calculation-only, calculation and language, manual tasks (i.e., pointing), and visuospatial tasks. The localizationalist view predicts that, like finger gnosis, all co-located functions such as left–right orientation and graphia, should be equally well correlated with math ability. This prediction, however, is not borne out. In contrast to finger gnosis, these co-located functions are uncorrelated or only weakly correlated with math ability (Noël, 2005). Importantly, on the localizationist view, there is no direct causal link between finger gnosis and math ability.

### FUNCTIONALIST VIEW

On the functionalist view, finger gnosis is related to math ability because fingers are used in the course of math development to represent quantities and perform counting and arithmetic procedures (Butterworth, 1999)^1^. Thus, the representations of fingers and of numbers become linked developmentally. According to this view, the comorbidity of finger agnosia and acalculia as well as the relation between finger gnosis and math in normally developing children arise because the representation of numbers is not only co-located with, but also linked to, the representation of fingers. The functionalist view predicts that facility in finger use (e.g., finger agility/fine-motor ability) should also predict math ability. This is in part because on the functionalist view there is a direct causal link between finger gnosis and math ability and, moreover, this link is formed experientially in the course of normal development. The precise motor control required to use the fingers to represent quantities and perform counting and arithmetic procedures is vital for the development of numeracy (Butterworth, 1999). Thus, it follows that children who can more easily use their fingers would form a stronger association between finger and number. Consistent with the functionalist view, fine motor deficits are associated with counting and calculation deficits (Barnes et al., 2005), though importantly not with deficits in the strength of number representations. Despite considerable variability in finger agility in typically developing children, finger agility is uncorrelated or only weakly correlated with performance on tasks assessing the strength of numerical representations – in contrast to finger gnosis (Penner-Wilger et al., 2007; Penner-Wilger et al., submitted).

### REDEPLOYMENT VIEW

In Penner-Wilger and Anderson (2008), we briefly outlined an alternative view of the relation between fingers and math that used as its base the massive redeployment hypothesis of brain evolution (Anderson, 2007a,b,c, 2008, 2010; Anderson and Penner-Wilger, 2013). Here we further develop the *redeployment view* of the relation between fingers and number.

The massive redeployment hypothesis (MRH) is both a theory about the functional topography of the cortex, and an account of how it got that way. According to MRH, neural circuits evolved for one cognitive or behavioral task (henceforth *use*) are frequently exapted for later uses when they perform a low-level function, or *cognitive working *that is useful in multiple task contexts. Cognitive workings are low-level operations that are performed by small, typically local anatomical circuits (Bergeron, 2008). As such, workings are neither consciously available nor describable at the higher level of psychological vocabulary. Multiple workings, in concert, contribute to higher-level cognitive *uses*.^2^ According to MRH, a typical brain area will contribute to many cognitive uses, across domains, performing the same working across these uses (Anderson, 2010). That is, one mechanism of cognitive evolution is analogous to component re-use in software engineering. Components originally developed to serve a specific purpose are frequently re-used in later software packages. The new software may serve a purpose very different from the software for which the component was originally designed, but may nevertheless require some of the same low-level computational workings (e.g., sorting). Thus, efficient development dictates re-use of existing components where possible. Note that in such re-use, the component just does whatever it does (e.g., sorts lists) for all the software packages into which it has been integrated, even if that computational working serves a very different high-level purpose, or use, in each individual case.

Two of the tenets of MRH (Anderson, 2010) are particularly relevant to the goals of this section. First, each brain area is typically redeployed in support of multiple cognitive uses both within and across domain boundaries. Second, redeployed areas have the same working in each of the functional complexes they support. Anderson (2007b, p. 339) uses the analogy of “finding the right letter to go into a box on a (multidimensional) crossword puzzle” to describe the task of determining a shared cognitive working. Thus, knowing the many cognitive uses that a brain area supports will help to determine what that brain area contributes to the many anatomico-functional complexes that it supports.

Preliminary investigations – generally involving data-mining a large collection of brain imaging experiments – have uncovered evidence for four specific predictions made by MRH. First, any given brain area is typically redeployed in support of many cognitive uses, and such redeployment will not respect traditional domain boundaries (that is, brain areas are not domain-restricted entities). Second, differences in domain uses will be accounted for primarily by differences in the way brain areas cooperate with one another, rather than by differences in which brain areas are used in each domain. Third, more recently evolved cognitive uses will utilize more, and more widely scattered brain areas. And fourth, evolutionarily older brain areas will be deployed in more cognitive uses. See Anderson (Anderson, 2007c, 2008, 2010) and Anderson and Penner-Wilger (2013) for details of the methods and results.

#### How redeployment explains the observed relation

**FIGURE 1:**
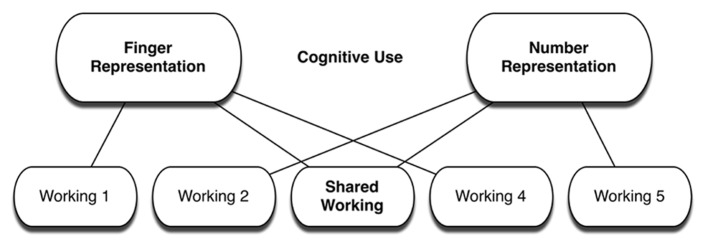
**Illustration of redeployment view of the relation between finger and number representations.**>

In line with the general findings of MRH, we propose that one of the neural circuits integrated into the functional complex supporting finger gnosis is also part of the functional complex supporting the representation of number. That is, one of the functional circuits originally evolved for finger representation has since been redeployed to support the representation of number and now serves both uses. Alternatively, the functional circuit may have originally evolved for a third use and been redeployed for both finger and number representation. Regardless, on the redeployment view, finger gnosis is related to math ability because part of the functional complex for number representation overlaps with the functional complex for finger representation. Thus, finger and number share a common neural circuit – a circuit that performs a shared working that supports both sorts of representation (see **Figure 1**). The comorbidity of finger agnosia and acalculia as well as the relation between finger gnosis and math in normally developing children arise from the shared neural circuit used for both representations.

The redeployment view of the relation between fingers and number is not a localizationist view. On the redeployment view, finger and number representations are not just neighboring neural functions on a correlated developmental trajectory; rather, they share a common neural substrate forming part of the neural complex supporting each function. Nor is the redeployment view a functionalist view. Importantly, on the redeployment view the connection between finger and number does not rest on the experienced use of the actual fingers to represent numerosities and perform arithmetic procedures, though it might suggest reasons we find it natural to use the fingers in this way, and thus might partially explain the finding that finger-counting strategies spontaneously appear at an early age for children in almost all cultures (Butterworth, 1999).

In sum, the primary claim of the redeployment view is that the functional complexes supporting finger and number representation share some neural circuitry. The next section outlines some specific predictions arising from this claim, and evaluates existing empirical evidence for those predictions.

At this point, some readers will surely be wondering: exactly what cognitive/computational working might account for the deployment of the shared circuit in these ostensibly very different uses? Exploring this specific question with the care it deserves would take us very far afield, for in fact the circuit in question is used not just in finger and number representation, but in a large number of other tasks, including word generation (e.g., Frankenstein et al., 2001), task-switching (e.g., Cools et al., 2004), response inhibition (e.g., Chikazoe et al., 2007), and more. However, we can report here that our preliminary analysis – including the construction and testing of a cross-domain computational model of the shared circuit using Nengo (Eliasmith et al., 2012) – suggests that an *array of pointers *has the required computational properties to contribute to both number and finger representation, as well as to the other tasks that appear to rely in part on this shared circuit. An array is a computational structure that offers ordered storage, while a pointer offers the capacity to index both different memory locations and data types. How such a computational structure serves the multiple uses supported by the circuit, how we approach the modeling of its function, how spiking neurons can in fact implement such a working, and why the results seem to favor this particular hypothesized working are discussed in detail in Penner-Wilger et al. (in preparation). For now, we hope that this brief outline offers the reader some idea of the kinds of things workings can be, both in general, and in this particular case.

Finally, it should be noted that Dehaene (2005) and Dehaene and Cohen (2007) outlines a view that, like MRH (Anderson, 2007a,c, 2010), takes as its base the mechanism of exaptation – whereby features (or functions) that evolved by selection for one purpose were later adapted to a new purpose. The explanatory goal of the neuronal recycling theory is to determine how humans acquire cultural tools such as reading and arithmetic. One notable difference between the two views is the time course of the mechanisms involved. On the neuronal recycling theory, re-use happens over the course of development whereas the re-use in MRH happens over the course of evolution (for a more detailed comparison of the two views, see Anderson, 2010). The two views need not be in conflict, however, because both evolutionary and developmental mechanisms reasonably contribute to novel cognitive functions. Dehaene, however, does not apply neuronal recycling to explain the relation between finger and number representation. While it would be interesting to explore the possible reasons for this, that will be left to a future paper.

## EVIDENCE AND PREDICTIONS FOR THE REDEPLOYMENT VIEW

If the relation between finger gnosis and math arises because part of the neural circuit responsible for the representation of fingers has been redeployed in support of the representation of number and now supports both uses then the following predictions should be borne out:

(1) Brain regions associated with the representation of fingers should be activated during tasks requiring the representation of number.
(2) Damage/disruption of the neural substrate should affect both finger gnosis and tasks requiring the representation of number.
(3) There should be measurable interference between tasks involving finger gnosis and tasks involving number representation, insofar as these would be competing for the same neural resource.
(4) Individuals with intact finger gnosis, who could not or did not use their fingers to represent quantities during development, should nevertheless show activation in the finger circuit during tasks requiring the representation of number.

### EVIDENCE FOR PREDICTION 1

Brain regions associated with the representation of fingers are activated during tasks requiring the representation of number. Prediction 1 would differentiate redeployment from localization, given adequate precision; however, it does not differentiate the redeployment view from the functionalist view, except in very young children. On both the functionalist and redeployment views, the representation of fingers and numbers are linked, with the key difference being the experiential requirement in the functionalist view.

There is strong empirical support for Prediction 1. Regions associated with finger representation within the left parietal lobe are activated during a variety of mathematical tasks: a region in the premotor strip (left precentral gyrus; Dehaene et al., 1996; de Jong et al., 1996; Jancke et al., 2000; Pesenti et al., 2000; Kuhtz-Buschbeck et al., 2003; Numminen et al., 2004; Pinel et al., 2004; Venkatraman et al., 2005; Liu et al., 2006) in the left angular gyrus (Göbel et al., 2004; Liu et al., 2006), and in the horizontal section of the intraparietal sulcus and in the posterior section of the superior parietal lobule (Andres et al., 2012). Zago et al. (2001) found activation of a finger-representation circuit in the left parietal lobe during adults’ performance of basic arithmetic. Increased activation was observed in the premotor strip at the coordinates associated with finger representation during multiplication performance compared to a digit reading condition. Andres et al. (2007), using transcranial magnetic stimulation over the left M1 hand area to measure changes in corticospinal excitability, found that hand motor circuits were activated during adults’ number processing in a dot counting task. Both sets of authors speculated that the activation might represent a developmental trace consistent with the functionalist view. The findings, however, are equally consistent with the redeployment view that part of the circuit responsible for the representation of fingers was redeployed in the representation of number.

In summary, across a variety of number and finger tasks, functional imaging studies have shown overlapping activation in parietal regions (Andres et al., 2007, 2012). Thus, the finding that brain regions associated with the representation of number and fingers are co-activated is robust, consistent with the functionalist and redeployment views. It remains possible, however, that future increases in the accuracy of functional imaging will eventually produce evidence favoring the localizationist view.

### EVIDENCE FOR PREDICTION 2

Damage/disruption affects both finger gnosis and tasks requiring the representation of number. Prediction 2 is again inconsistent with the localizationist view, yet it does not differentiate between the redeployment and functionalist views. Studies where disruption was induced using either repetitive transcranial magnetic stimulation (rTMS) or direct cortical stimulation provide converging evidence that disruption in the left angular gyrus affects both finger gnosis and tasks requiring the representation of number.

Rusconi et al. (2005) used rTMS applied to parietal sites to determine if there was a common neural substrate between number and fingers. In a series of experiments, they found that rTMS over the left angular gyrus disrupted both magnitude comparison and finger gnosis in adults. Roux et al. (2003) using direct cortical stimulation also found a site in the left angular gyrus that produced both acalculia and finger agnosia. Thus, consistent with the redeployment and functionalist views, stimulation of the left angular gyrus across methods has been found to disrupt finger gnosis along with number comparison and calculation.

### EVIDENCE FOR PREDICTION 3

Prediction 3 is that there should be measurable interference between tasks involving finger gnosis and tasks involving number representation, as these would be competing for the same neural resource. Two methods would allow for the testing of this hypothesis: a dual task paradigm or injection of noise into the system. As an example of noise injection, electrical stimulation of the fingers (but not of various locations on the forearm) might impact performance on mathematical tasks.

A recent study by Brozzoli et al. (2008) is relevant here. In this experiment, participants were asked to press a foot pedal whenever either their thumb or little finger on the right hand was electrically stimulated. Just prior to the onset of this tactile stimulus, participants were shown one of four numbers: 1, 2, 4, or 5. With the hand oriented palm down, response times to the tactile stimulus of the thumb were positively correlated with the magnitude of the number, while response times to the tactile stimulus of the little finger were negatively correlated with the magnitude. With the hand oriented palm up, the results were just the opposite. That is, the task interaction in this case appears to be spatially mediated.

Not only does this demonstrate the predicted cross-talk between finger and number circuits, the result is somewhat awkward to explain on the functionalist view. Given the important role of experience in setting up the finger–number associations, the functionalist view would presumably predict fairly strong associations between individual fingers and specific numbers, induced by the particular counting procedures typically employed. In this case, participants all employed a counting procedure starting with the thumb for “1,” and continuing in order down the hand to the little finger (“5”). On the functionalist view, one would not expect these associations to be modulated by hand orientation.^3^

In contrast, the finding is compatible with (although not specifically predicted by) the redeployment view. This is because on the redeployment view, there need be no strong association between individual fingers and specific numbers. The entwinement of finger and number representations is not established by experience, but is the result of the fact that the two functional complexes share a neural circuit that does something useful for both. Even if what is being shared is a particular representational storage resource such as an array – as was proposed by Penner-Wilger and Anderson, 2008 – this would not suggest strong associations between individual fingers and numbers.

Because on the redeployment view any given neural resource is being utilized by potentially many different functional complexes, understanding the functional relationship requires a strong distinction between the representation itself – what is being stored – and the representation consumer that is using the thing stored (Millikan, 1984). The content of a representation depends both on the intrinsic properties of the representation and on the details of the mechanism that treats the representation as having significance. For a simple example, consider the following representation: 1001. Depending on the context, and on the assumptions of the interpreter, that representation can be taken to have the same content as the English phrase “one thousand and one” or as the Arabic numeral 9.^4^ It could conceivably also have alphabetic, numerological, or iconographic content, or be an instruction set for a Turing machine. The point is: on the redeployment view, the meaning of whatever representation might be stored in that resource would depend on the representation consumer, and this meaning could vary greatly depending on the needs of the functional complexes incorporating the resource.

Note that if two tasks *are* using the same representational resource, they will interfere with one another only when the representation for one use is incongruent with the representation for the other concurrent use. Thus if by chance a finger stimulus produced activation consistent with the standing number representation, there would be no interference. This is relevant because counting on the fingers, during which process one successively stimulates (touches, moves, etc.) fingers, is a real-world instance of a finger stimulation task that by design produces representations in the shared representational resource that can provide accurate information to consumers in both complexes: I know I have reached 7 because I have just touched *this* finger. This suggests a further implication. Although the details of the procedure one can use to count on one’s fingers – which fingers are touched in which order with what meaning – are highly various (Bender and Beller, 2012), even theoretically arbitrary, the set of such procedures that can produce representations that would be accurate in both domains would be constrained by the representation consumers in both domains; not every procedure will produce representations compatible with the available consumers (see Fischer, 2008 and Bender and Beller, 2012 for evidence that different counting habits can indeed impact psychological outcomes). Thus, on the redeployment view, there could exist a set of self-interfering finger-based counting procedures. The complementary implication is that there would be a set of procedures that are more natural and/or easier to acquire, insofar as they produce representations consistent with existing consumers, although this would clearly not be the only aspect of the procedure relevant to ease of acquisition and use (Bender and Beller, 2012).

Discovering self-interfering counting procedures would seem to count against both the localizationist view and the functionalist view. If the intertwining of representations is the result of experience, then there need be no *a priori* limit on the nature of the procedure that would cause the intertwining; and no consistent procedure that produced intertwining could be self-interfering. Likewise with the complementary implication: if any such procedure could be learned, then there is no specific theoretical reason that one should be easier than another.

**FIGURE 2:**
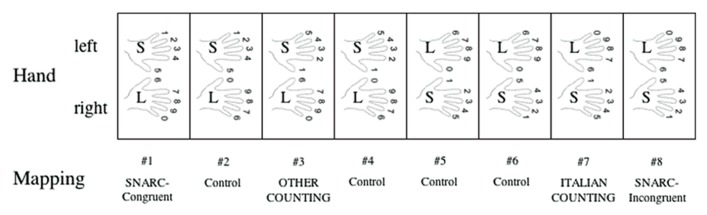
**The various finger-digit mappings tested in [Bibr B26]**.

Di Luca et al. (2006) report some results relevant to these predictions. In this experiment, participants were taught various finger-digit mappings (two per participant; see **Figure 2**). They then showed the participants numbers on a computer screen, and asked them to press the key underneath the matching finger.

That participants were able to do this task with very little training suggests that it is indeed possible to teach alternate systems in a short time. However, there were significant differences in the number of errors participants made with different mappings. Naturally, mapping 7 – identical to mapping from the participants’ primary counting procedure – showed very few errors, but interestingly, there were no significant differences in the number of errors made for mappings 1, 7, and 8, even though mapping 1 has no overlap with 7, and a five digit overlap with 8. Even more interesting, mappings 2–6 induced significantly more errors than did 1, 7, and 8, even though some of them (e.g., 5) have a five digit overlap with 7, and others (e.g., 6 and 2 have five digit overlaps with 8 and 1). Thus, it does not appear that any strong associations induced by the familiar counting procedure are responsible for making mappings 2–6 harder than the rest. There must be some other explanation, and limits imposed by existing representation consumers – as predicted by redeployment – offer one plausible option.

This evidence is more suggestive than definitive, but it certainly seems an intriguing line of research that could push both models in new directions. For instance, the discovery of a counting procedure or finger–number mapping that did not just reduce performance, but resulted in systematic errors consistent with the mismatch between the procedure and the inferred properties of a representation consumer could be strong evidence for the redeployment view.

In general, which outcomes in interference experiments would, and would not, be consistent with the functionalist view might depend in part on the nature and timeline of the hypothesized intertwining of representations that occurs during development. The strongest interpretation of intertwining is that number representations and the finger sense become inextricably linked, even coming to share the same neural resources. If this is the hypothesis, then perhaps redeployment and functionalism cannot be distinguished based on interference studies, since they would then posit the same underlying neural relationship, and differ only with respect to the account of how that relationship came about. However, there are weaker versions of intertwining that are consistent with the original model. For instance, one might expect that number representations would come to depend on the finger sense, but not the reverse. Such a model might be somewhat more plausible developmentally speaking, as although it may be typical to use the fingers whenever one is doing mathematics, it would certainly be atypical to think of mathematics whenever one is using the fingers. At the very least, the various possibilities to be entertained in designing interference experiments suggest the need for a clarification of the functionalist model.

### EVIDENCE FOR PREDICTION 4

Prediction 4 is that individuals with intact finger gnosis, who could not or did not use their fingers to represent quantities during development, will nevertheless show activation in the finger circuit during tasks requiring the representation of number. Prediction 4 is the key in distinguishing the redeployment view from the functionalist view. Special populations may play a crucial role in testing this prediction.

If the relation between fingers and number is a functional one, then the use of ones’ fingers to represent numerosities and perform counting and arithmetic procedures would be a necessary element in the development of numeracy. We have already found that finger gnosis in a sample of normally achieving children is more highly correlated with numeracy and calculation skills than is finger agility. This finding is apparently at odds with the functionalist view, but is consistent with the redeployment view that the connection between fingers and number does not rest on the experiential use of fingers to represent number.

One possible route for further investigation would involve imaging experiments using participants from cultures without the practice of using fingers in mathematical contexts. It has been reported, for instance, that various populations including the Amazonian Pirahã and children in Australia’s Northern Territory do not use their fingers in support of mathematical tasks (Everett, 2005; Butterworth et al., 2011; Bender and Beller, 2012). The redeployment view would nevertheless predict the use of a common brain region during both finger gnosis and number representation tasks.

Another route would involve investigations using children presenting specific developmental disorders. Children with spina bifida have both finger agnosia and poor finger agility co-morbid with significant mathematical difficulties (Bannister and Tew, 1991; Barnes et al., 2005). This finding has been taken as evidence for a functional role of fingers in mathematical development, as children with spina bifida would have difficulty using their fingers to form a functional/developmental link with number. However, as this population also has disrupted finger gnosis, the finding is likewise consistent with the redeployment view.

In contrast, children with developmental coordination disorder (DCD) have poor finger agility, but most have preserved finger gnosis (Cermak and Larkin, 2001; Hamilton, 2002). Thus, children with DCD are ideally suited as a population with which to test the redeployment view against the functionalist view. Approximately 6% of children meet the criteria for DCD outlined in the DSM-IV. On the redeployment view, we predict that children with DCD and preserved finger gnosis will show activation in the finger circuit during tasks requiring the representation of number such as magnitude comparison. We are currently designing an imaging experiment to test this prediction in a population of children with DCD.

As a first indication of support for the redeployment view, DCD is not generally comorbid with mathematical difficulties (Cermak and Larkin, 2001). Thus, despite motor problems limiting the ability to use the fingers to represent numerosities, the representation of number appears unaffected in children with DCD. This finding is consistent with the redeployment view, but presents difficulties for the functionalist view. On the redeployment view, children with DCD might be expected to show some deficits with arithmetic, given a functional role for the fingers in the development of counting and arithmetic procedures, but such deficits would not be expected to impact numerical representation. On the functionalist view, the use of fingers is necessary for the development of both numerical representations and arithmetic procedures. Hence, on the functionalist view, children with DCD would be expected to show widespread math disabilities as seen in children with spina bifida.

### SUMMARY OF AVAILABLE EVIDENCE

In summary, two of the four predictions that arise from the redeployment view are well supported by empirical evidence: (1) brain regions associated with the representation of fingers are activated during tasks requiring the representation of number and (2) damage/disruption of the neural substrate affects both finger gnosis and tasks requiring the representation of number. Prediction 3, that there should be measureable interference between tasks involving finger gnosis and tasks involving number representation has yet to be systematically investigated, although we review some existing evidence that appears to point in that direction. There is also suggestive evidence for Prediction 4, that individuals with intact finger gnosis, who could not or did not use their fingers to represent quantities during development, will nevertheless show activation in the finger circuit during tasks requiring the representation of number.

The data reviewed above strongly favors the first two predictions, which are inconsistent with the localizationist view. Even so, supporters of localization might question the strength of the results, since the spatial resolution of the imaging methods employed in some of those studies is poor enough that it is theoretically possible for circuits that are in fact segregated to appear to overlap. Thus, experiments using methods with greater ability to differentiate between local circuits may be called for. For instance, in investigating the hypothesized overlap between motor control and language processing, Glenberg et al. (2008) employed a neural attenuation paradigm wherein a long session of repeated movements induced changes in the responses of neural populations that affected processing in a language task.

Although the localizationist view appears to be ruled out, pending further evidence to the contrary, support for the redeployment view could not be conclusively distinguished from that for the functionalist view on the basis of evidence for Predictions 1–3. Thus, support for Prediction 4 would be the crucial evidence to conclude that the relation between fingers and number is not functional. We provided evidence consistent with Prediction 4 and outlined further empirical tests of the redeployment view. That said, the redeployment and functionalist views need not ultimately be in conflict – it is likely that there is both a developmental and an evolutionary story to tell about the overall relation between fingers and mathematical ability.

## CONCLUSION

This paper elaborated a novel hypothesis regarding the observed predictive relation between finger gnosis and mathematical ability. In brief, we suggested that these two cognitive capacities have overlapping neural substrates, as the result of the re-use (“redeployment”) of part of the finger gnosis circuit for the purpose of representing number. On balance, the evidence seems to favor the redeployment account of the relation, over the functionalist and localizationist accounts. Of course, more research will be needed, and we have indicated some of the paths such research might follow.

It is important to reiterate that on the redeployment view, the neural circuitry shared between finger gnosis and number representation forms only *one* part of the functional complex necessary for number representation. In MRH, existing neural circuits are redeployed for new uses and combined to support new capacities. Along with the neural circuit shared with finger gnosis, additional neural circuits (with additional abstract functional capacities) are expected to combine in support of the capacity for number representation.

If redeployment is the right framework for understanding the relation between finger gnosis and math, the natural next question is: what could the brain region in question be *doing* for each of these apparently quite different domains? An important implication of the redeployment view is that such questions should be approached using a cross-domain structure-function mapping methodology, as discussed above. Using this methodology, we have examined 2164 imaging experiments from the BrainMap and NICAM databases (Laird et al., 2005; Anderson et al., 2010) to guide and constrain the answer to what the working of this circuit is that allows it to support tasks in such apparently different cognitive domains (Penner-Wilger and Anderson, 2011; Anderson and Penner-Wilger, 2013; Penner-Wilger et al., in preparation).

## Conflict of Interest Statement

The authors declare that the research was conducted in the absence of any commercial or financial relationships that could be construed as a potential conflict of interest.

## Footnotes

1 It should be noted that this use of the term “functionalist” is not in keeping with its current use in cognitive science, although it is at least compatible with its use in the history of psychology (e.g., functionalism) more broadly. In any event, the localizationist/functionalist distinction is taken from the existing literature on finger gnosis and math ability, and so we stick with that usage, here (Marinthe et al., 2001; Noël, 2005).

2 The vocabulary of workings and uses results from the fact that terms like *function* and *role* were used inconsistently (and in fact used by different theorists in opposite ways) within the cognitive science literature (Bergeron, 2008).

3 It would also be hard to explain on any more general theory of association by experience, such as Pulvermüller (2005) proposes to account for the sensory-motor grounding of lexical concepts.

4 Hence the old joke: there are 10 kinds of people in the world: those that understand binary and those that do not.

## References

[B1] Anderson M. L. (2007a). The massive redeployment hypothesis and the functional topography of the brain. *Philos. Psychol.* 2 143–174 10.1080/09515080701197163

[B2] Anderson M. L. (2007b). Massive redeployment, exaptation, and the functional integration of cognitive operations. *Synthese* 159 329–345 10.1007/s11229-007-9233-2

[B3] Anderson M. L. (2007c). Evolution of cognitive function via redeployment of brain areas. *Neuroscientist* 13 13–21 10.1177/1073858406294706 17229971

[B4] Anderson M. L. (2008). Circuit sharing and the implementation of intelligent systems. *Conn. Sci.* 20 239–251 10.1080/09540090802413202

[B5] Anderson M. L. (2010). Neural reuse: a fundamental organizational principle of the brain. *Behav. Brain Sci.* 33 245–266 10.1017/S0140525X10000853 20964882

[B6] Anderson M. L. Brumbaugh J. Şuben A. (2010). “Investigating functional cooperation in the human brain using simple graph-theoretic methods,” in *Computational Neuroscience* eds Chaovalitwongse A. Pardalos P. M. Xanthopoulos P. (Berlin, Germany: Springer) 31–42

[B7] Anderson M. L. Penner-Wilger M. (2013). Neural reuse in the evolution and development of the brain: evidence for developmental homology? *Dev. Psychobiol.* 55 42–51 10.1002/dev.21055 22711453

[B8] Andres M. Michaux N. Pesneti M. (2012). Common substrate for mental arithmetic and finger representation in the parietal cortex. *Neuroimage* 62 1520–1528 10.1016/j.neuroimage.2012.05.047 22634854

[B9] Andres M. Seron X. Oliver E. (2007). Contribution of hand motor circuits to counting. *J. Cogn. Neurosci.* 19 563–576 10.1162/jocn.2007.19.4.563 17381248

[B10] Bannister C. M. Tew B. (1991). *Current Concepts in Spina Bifida and Hhydrocephalus.* Cambridge: Cambridge University Press

[B11] Barnes M. A. Smith-Chant B. L. Landry S. (2005). “Number processing in neurodevelopmental disorders: spina bifida myelomenigocele,” in *Handbook of Mathematical Cognition* ed. Campbell J. I. D. (New York, NY: Psychology Press) 299–314

[B12] Baron I. S. (2004). *Neuropsychological Evaluation of the Child*. New York, NY: Oxford University Press

[B13] Bender A. Beller S. (2012). Nature and culture of finger counting: diversity and representational effects of an embodied cognitive tool. *Cognition* 124 156–182 10.1016/j.cognition.2012.05.005 22695379

[B14] Bergeron V. (2008). *Cognitive Architecture and the Brain: Beyond Domain-Specific Functional Specification*. Unpublished doctoral dissertation, University of British Columbia Vancouver, British Columbia, Canada

[B15] Brozzoli C. Ishihara M. Göbel S. M. Salemme R. Rossetti Y. Farnè A. (2008). Touch perception reveals the dominance of spatial over digital representation of numbers. *Proc. Natl. Acad. Sci. U.S.A.* 105 5644–5648 10.1073/pnas.0708414105 18385382 PMC2291099

[B16] Butterworth B. (1999). *What Counts – How Every Brain is Hardwired for Math.* New York, NY: The Free Press

[B17] Butterworth B. Reeve R. Reynolds F. (2011). Using mental representations of space when words are unavailable: studies of enumeration and arithmetic in indigenous Australia. *J. Cross Cult. Psychol.* 42 630–638 10.1177/0022022111406020

[B18] Cermak S. A. Larkin D. (2001). *Developmental Coordination Disorder*. Albany, NY: Delmar

[B19] Chikazoe J. Konishi S. Asari T. Jimura K. Miyashita Y. (2007). Activation of right inferior frontal gyrus during response inhibition across response modalities. *J. Cogn. Neurosci.* 19 69–80 10.1162/jocn.2007.19.1.69 17214564

[B20] Cools R. Clark L. Robbins T. W. (2004). Differential responses in human striatum and prefrontal cortex to changes in object and rule relevance. *J. Neurosci.* 24 1129–1135 10.1523/JNEUROSCI.4312-03.2004 14762131 PMC6793591

[B21] Dehaene S. (2005). “Evolution of human cortical circuits for reading and arithmetic: the “neuronal recycling” hypothesis,” in *From Monkey Brain to Human Brain* eds Dehaene S. Duhamel J. R. Hauser M. Rizzolatti G. (Cambridge, MA: MIT Press) 133–157

[B22] Dehaene S. Cohen L. (2007). Cultural recycling of cortical maps. *Neuron* 56 384–398 10.1016/j.neuron.2007.10.004 17964253

[B23] Dehaene S. Piazza M. Pinel P. Cohen L. (2003). Three parietal circuits for number processing. *Cogn. Neuropsychol.* 20 487–506 10.1080/02643290244000239 20957581

[B24] Dehaene S. Tzourio N. Frak V. Raynaud L. Cohen L. Mehler J. (1996). Cerebral activations during number multiplication and comparison: a PET study. *Neuropsychologia* 34 1097–1106 10.1016/0028-3932(96)00027-9 8904747

[B25] de Jong B. M. van Zomeren A. H. Willemsen A. T. M Paans A. M. J. (1996). Brain activity related to serial cognitive performance resembles circuitry of higher order motor control. *Exp. Brain Res.* 109 136–140 10.1007/BF00228634 8740216

[B26] Di Luca S. Granà A. Semenza C. Seron X. Presenti M. (2006). Finger–digit compatibility in Arabic numeral processing. *Q. J. Exp. Psychol.* 59 1648–1663 10.1080/17470210500256839 16873114

[B27] Eliasmith C. Stewart T. C. Choo X. Bekolay T. DeWolf T. Tang Y. (2012). A large-scale model of the functioning brain. *Science* 338 1202–1205 10.1126/science.1225266 23197532

[B28] Everett D. L. (2005). Cultural constraints on grammar and cognition in Pirahã. *Curr. Anthropol.* 46 621–646 10.1086/431525

[B29] Fayol M. Barrouillet P. Marinthe C. (1998). Predicting arithmetical achievement from neuro-psychological performance: a longitudinal study. *Cognition* 68 B63–B70 10.1016/S0010-0277(98)00046-8 9818514

[B30] Fischer M. H. (2008). Finger counting habits modulate spatial–numerical associations. *Cortex* 44 386–392 10.1016/j.cortex.2007.08.004 18387569

[B31] Frankenstein U. N. Richter W. McIntyre M. C. Remy F. (2001). Distraction modulates anterior cingulate gyrus activations during the cold pressor test. *Neuroimage* 14 827–836 10.1006/nimg.2001.0883 11554801

[B32] Gerstmann J. (1940). Syndrome of finger agnosia, disorientation for right and left, agraphia, and acalculia. *Arch. Neurol. Psychiatry* 44 398–408 10.1001/archneurpsyc.1940.02280080158009

[B33] Gilandas A. Touyz S. Beumont P. J. V. Greenberg H. P. (1984). *Handbook of Neuropsychological Assessment*. Orlando, FL: Grune and Stratton

[B34] Glenberg A. M. Sato M. Cattaneo L. (2008). Use-induced motor plasticity affects the processing of abstract and concrete language. *Curr. Biol.* 18 R290–R291 10.1016/j.cub.2008.02.036 18397734

[B35] Göbel S. M. Johansen-Berg H. Behrens T Rushwort M. F. S. (2004). Response-selection-related parietal activation during number comparison. *J. Cogn. Neurosci.* 16 1536–1551 10.1162/0898929042568442 15601517

[B36] Hamilton S. S. (2002). Evaluation of clumsiness in children. *Am. Fam. Physician* 66 1435–1440 12408418

[B37] Jancke L. Loose R. Lutz K. Specht K. Shah N. J. (2000). Cortical activations during paced finger-tapping applying visual and auditory pacing stimuli. *Cogn. Brain Res.* 10 51–66 10.1016/S0926-6410(00)00022-7 10978692

[B38] Kuhtz-Buschbeck J. P. Mahnkopf C. Holzknecht C. Siebner H. R. Ulmer S. Jansen O. (2003). Effector-independent representations of simple and complex imagined finger movements: a combined fMRI and TMS study. *Eur. J. Neurosci.* 18 3375–3387 10.1111/j.1460-9568.2003.03066.x 14686911

[B39] Laird A. R. Lancaster J. L. Fox P. T. (2005). BrainMap: the social evolution of a functional neuroimaging database. *Neuroinformatics* 3 65–78 10.1385/NI:3:1:065 15897617

[B40] Liu X. Wang H. Corbly C. R. Zhang J. Joseph J. E. (2006). The involvement of the inferior parietal cortex in the numerical stroop effect and the distance effect in a two-digit number comparison task. *J. Cogn. Neurosci.* 18 1518–1530 10.1162/jocn.2006.18.9.1518 16989552

[B41] Marinthe C. Fayol M. Barrouillet P. (2001). “Gnosies digitales et développement des performances arithmétiques,” In *Troubles du calcul et dyscalculies chez l’enfant* ed. Van Hout et A. Meljac C. (Paris: Masson) 239–254

[B42] Millikan R. (1984). *The Language of Thought and Other Biological Categories*. Cambridge, MA: MIT Press

[B43] Noël M-P. (2005). Finger gnosia: a predictor of numerical abilities in children? *Child Neuropsychol.* 11 413–430 10.1080/09297040590951550 16306017

[B44] Numminen J. Schurmann M. Hiltunen J. Joensuu R. Jousmaki V. Koskinen S. K. (2004). Cortical activation during a spatiotemporal tactile comparison task. *Neuroimage* 22 815–821 10.1016/j.neuroimage.2004.02.011 15193610

[B45] Penner-Wilger M. (2013). Symbolic and non-symbolic distance effects in number comparison and ordinality tasks. *Paper Presented at the Canadian Society for Brain, Behaviour, and Cognitive Science Annual Meeting* Calgary, AB

[B46] Penner-Wilger M. Anderson M. L. (2008). “An alternative view of the relation between finger gnosis and math ability: redeployment of finger representations for the representation of number,” in *Proceedings of the 30th Annual Cognitive Science Society* eds Love B. C. McRae K. Sloutsky V. M. (Austin, TX: Cognitive Science Society) 1647–1652

[B47] Penner-Wilger M. Anderson M. L. (2011). “The relation between finger gnosis and mathematical ability: can we attribute function to cortical structure with cross-domain modeling?” in *Proceedings of the 33rd Annual Cognitive Science Society* (Austin, TX: Cognitive Science Society) 2445–2450

[B48] Penner-Wilger M. Fast L. LeFevre J. Smith-Chant B. L. Skwarchuk S. Kamawar D. (2009). “Subitizing, finger gnosis, and the representation of number,” in *Proceedings of the 31st Annual Cognitive Science Society* (Austin, TX: Cognitive Science Society) 520–525

[B49] Penner-Wilger M. Fast L. LeFevre J. Smith-Chant B. L. Skwarchuk S. Kamawar D. (2007). “The foundations of numeracy: subitizing, finger gnosia, and fine-motor ability,” in *Proceedings of the 29th Annual Cognitive Science Society* eds McNamara D. S. Trafton J. G. (Austin, TX: Cognitive Science Society) 1385–1390

[B50] Pesenti M. Thioux M. Seron X De Volder A. (2000). Neuroanatomical substrate of Arabic number processing, numerical comparison and simple addition: a PET study. *J. Cogn. Neurosci.* 12 461–479 10.1162/089892900562273 10931772

[B51] Pinel P. Piazza M. LeBihan D. Dehaene S. (2004). Distributed and overlapping cerebral representations of number size and luminance during comparative judgements. *Neuron* 41 983–993 10.1016/S0896-6273(04)00107-2 15046729

[B52] Pulvermüller F. (2005). Brain mechanisms linking language and action. *Nat. Rev. Neurosci.* 6 576–582 10.1038/nrn1706 15959465

[B53] Roux F-E. Boetto S. Sacko O. Chollet F. Tremoulet M. (2003). Writing, calculating, and finger recognition in the region of the angular gyrus: a cortical stimulation study of Gerstmann syndrome. *J. Neurosurg.* 99 716–727 10.3171/jns.2003.99.4.0716 14567608

[B54] Rusconi E. Walsh V. Butterworth B. (2005). Dexterity with numbers: rTMS over left angular gyrus disrupts finger gnosis and number processing. *Neuropsychologia* 43 1609–1624 10.1016/j.neuropsychologia.2005.01.009 16009243

[B55] Simon O. Mangin J. F. Cohen L. Le Bihan D. Dehaene S. (2002). Topographical layout of hand, eye, calculation, and language-related areas in the human parietal lobe. *Neuron* 33 475–487 10.1016/S0896-6273(02)00575-5 11832233

[B56] Venkatraman V. Ansari D Chee M. W. L. (2005). Neural correlates of symbolic and non-symbolic arithmetic. *Neuropsychologia* 43 744–753 10.1016/j.neuropsychologia.2004.08.005 15721187

[B57] Zago L. Pesenti M. Mellet E. Crivello F. Mazoyer B. Tzourio-Mazoyer N. (2001). Neural correlates of simple and complex mental calculation. *Neuroimage* 13 314–327 10.1006/nimg.2000.0697 11162272

